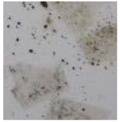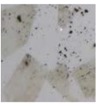# Melt Viscoelastic Assessment of Poly(Lactic Acid) Composting: Influence of UV Ageing

**DOI:** 10.3390/molecules23102682

**Published:** 2018-10-18

**Authors:** Vincent Verney, Audrey Ramoné, Florence Delor-Jestin, Sophie Commereuc, Marek Koutny, Geoffrey Perchet, Julien Troquet

**Affiliations:** 1 Institut de Chimie de Clermont Ferrand (ICCF), UMR 6296 Université Clermont Auvergne, CNRS, Sigma Clermont, ICCF, F-63000 Clermont-Ferrand, France; ramone.audrey@gmail.com (A.R.); florence.delor_jestin@sigma-clermont.fr (F.D.-J.); sophie.commereuc@sigma-clermont.fr (S.C.); 2 Faculty of Technology, Department of Environmental Engineering, Tomas Bata University in Zlin,762 72 Zlín, Czech Republic; mkoutny@ft.utb.cz; 3 Biobasic Environnement, Biopôle Clermont Limagne, 63360 Saint-Beauzire, France; geoffrey.perchet@laposte.net (G.P.); jtroquet@biobasicenvironnement.com (J.T.)

**Keywords:** rheology, PLA, biodegradation, photo-degradation

## Abstract

This study is devoted to the degradation pathway (bio, photo degradation and photo/bio) of Poly(Lactic acid) PLA polymers by means of melt viscoelasticity. A comparison was made between three PLA polymers with different microstructures (L, D stereoisomers). Biodegradability was determined during composting by burying the polymer films in compost at 58 °C. Melt viscoelasticity was used to assess the molecular evolution of the materials during the composting process. Viscoelastic data were plotted in the complex plane. We used this methodology to check the kinetics of the molecular weight decrease during the initial stages of the degradation, through the evolution of Newtonian viscosity. After a few days in compost, the Newtonian viscosity decreased sharply, meaning that macromolecular chain scissions began at the beginning of the experiments. However, a double molar mass distribution was also observed on Cole–Cole plots, indicating that there is also a chain recombination mechanism competing with the chain scission mechanism. PLA hydrolysis was observed by infra-red spectroscopy, where acid characteristic peaks appeared and became more intense during experiments, confirming hydrolytic activity during the first step of biodegradation. During UV ageing, polymer materials undergo a deep molecular evolution. After photo-degradation, lower viscosities were measured during biodegradation, but no significant differences in composting were found.

## 1. Introduction

Nowadays, the impact of domestic wastes on the environment is the focus of many researchers. During service life, it is well known that polymer materials age, mainly if they are exposed to UV light. The consequence is a decrease of the material properties leading to failure and waste production. The questions of the ecological treatment of these wastes have motivated many studies on biodegradable polymers [1,2,3].

Poly(lactic acid) is a good candidate to respond to this problem, because it is compostable [4,5,6] and bio-based [7]. PLA show properties comparable to other polymers like polyethylene or polystyrene [8]. Its particularity is its capacity to be easily hydrolyzed and thermo-degraded which confers it some compostable properties. PLA can be made from d-lactide and l-lactide [9]. In this way, PLLA and PDLA can be obtained. Commercial polymers are a mixture of d- and l-lactides, usually with a low d-content that is close to 10%. These different microstructures have been suspected to impact its biodegradation rate [10,11,12].

Biodegradation of organic compounds is usually quantified by material mineralization measurements. However, compost degradation is a complex phenomenon with a poorly understood mechanism [13,14,15]. Moreover, environmental biodegradation is difficult to control and reproduce adequately in lab conditions. Results often depend on temperature, micro-organism populations and humidity [16]. Methods have been standardized, but they do not consider the suns impact, despite UV possibly alters the polymer structure. During UV ageing, polymer materials undergo a deep molecular evolution characterized by chain scissions, chain crosslinking or more usually a competition between both mechanisms [17]. In the case of chain crosslinking, some papers share some doubts about the biodegradable character of biodegradable polymers after aging, and try to correlate the decrease of biodegradation rates with a gel content increase [18,19]. In the case of semi-crystalline polymers, chain scissions can impact crystalline morphologies, and crystallization rates through a reduction of molecular weight [20,21]. It has also been demonstrated there is some decrease in biodegradability due to these crystalline changes [22,23].

The goal of this study was to use melt viscoelasticity to assess the molecular evolution of the materials under UV exposure and biodegradation [24]. We have used this methodology to investigate the kinetics of the molecular weight decrease during biodegradation, before and after photo-degradation by a decrease of Newtonian viscosity. During biodegradation tests, samples (previously photo-aged or not) were analyzed by melt viscoelasticity.

Biodegradation tests have been carried out in compost, with cellulose (blotting paper) as a reference material. PLA compression molded films (≠100 μm) were exposed to UV radiation under accelerated weathering conditions in a SEPAP Atlas oven. A comparison was made between the non-photo-aged and the photo-aged polymer behaviors during biodegradation using melt viscoelasticity.

## 2. Experimental Section

### 2.1. Materials and Methods

#### 2.1.1. Polymers Used and Sample Preparation

The three PLAs used were purchased from Naturplast: a 100% poly(l-lactic acid), a 100% poly(d-lactic acid) and a commercial PLA named PLA 4042D. It is an optical copolymer of predominantly L isomer. 6% D-content in PLA 4042D was determined by polarimetry [25]. Pellets were dissolved in chloroform and specific rotation ${\left({\left[\alpha \right]}_{D}^{25}\right)}_{PLA}$ was measured with a polarimeter. *D*% content can be calculated as follows:

(1) $$D\%=\frac{{\left({\left[\alpha \right]}_{D}^{25}\right)}_{PLLA}-{\left({\left[\alpha \right]}_{D}^{25}\right)}_{PLA}}{2{\left({\left[\alpha \right]}_{D}^{25}\right)}_{PLLA}}\times 100\%$$

The specific rotation of pure PLLA [26] is ${\left({\left[\alpha \right]}_{D}^{25}\right)}_{PLLA}$ = −156°.

The most important physical properties are listed in Table 1.

Polymers films were obtained by hot compression molding under 200 bar of pressure, during 1 min 30 s at a temperature higher than melting temperature, 180 °C. Films below 130 μm of thickness were used for following experiments.

#### 2.1.2. Analytical Methods

Fourier transform infrared absorption spectra were obtained in transmission mode using a Thermo Scientific Nicolet 6700 FTIR spectrometer and OMNIC software (Thermo fisher company, Waltham, MA, USA). The spectra were collected with 32 accumulations at a resolution of 4 cm^−^^1^ in the 400–4000 cm^−^^1^ range.

#### 2.1.3. Melt Rheology

Molecular changes were monitored by melt viscoelasticity experiments in oscillatory shearing mode using a rotational controlled strain rheometer (ARES/Rheometric from Scientific-TA Instruments) equipped with parallel plate geometry (diameter 25 mm and gap 1 mm).

For each sample, strain amplitude values were checked to be sure they worked within the linear viscoelastic region. The strain amplitude were fixed and used for measuring the dynamic stresses during oscillatory shearing at different frequencies. Frequency sweeps extending from 0.1 to 100 rad/s were performed under dry air flow at 180 °C.

Oscillatory frequency sweeps test consisted of measuring the resulting stress σ*(t) for an applied sinusoidal strain γ*(t). The stress σ(t) and strain speed $\overset{\cdot }{\gamma }\left(t\right)$ ratio equals the complex dynamic viscosity η*(ω):

(2) $$\eta *\left(\omega \right)=\frac{\sigma *\left(t\right)}{\overset{\cdot }{\gamma }\left(t\right)}\;$$

η*(ω) = η′(ω) − j η″(ω)(3)

where η′(ω) is the loss viscosity (stress-strain out of phase component) and η″(ω) the storage viscosity (stress-strain in phase component). “j” is the complex number such as j^2^ = −1.

**Scheme 1:**
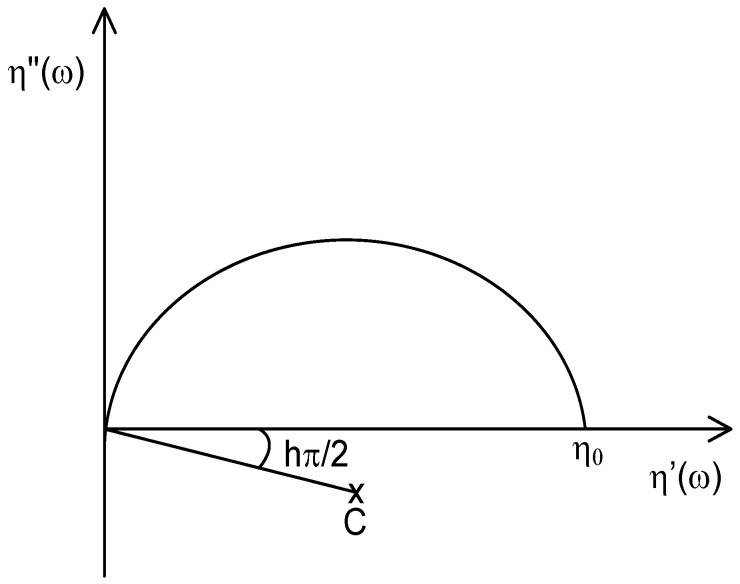
Complex plane representation: Cole–Cole plots.

Viscoelastic data can be plotted in the complex plane: 

This representation, also called Cole-Cole plot, consists of plotting the imaginary part (η″(ω): storage viscosity) of the complex viscosity (η*) against the real one (η′ (ω): loss viscosity) [27]. In an entangled polymer melt, the relaxation behavior gives rise to a semi-circular variation of the experimental measurements during oscillatory shearing. It is used to determine the Newtonian zero-shear viscosity η_0,_ which corresponds to the intersection between the extrapolated semi-circle and the X-axis (see Scheme 1). This value is proportional to the molecular weight M_w_ according to a α power-law relationship.

η_0_ = K∙(M_w_) ^a^ (4)

K and a are constants. The “a” value was determined to be 3.4–3.6 [28,29].

#### 2.1.4. Biodegradation in Compost

Compost made from wood and other green wastes came from a compost station in Puy-de-Dôme (France). Tests were performed at 58 °C with air flow and 50% of relative humidity. Compost was sieved to 3 mm before use. Dried content (%m_d_) was 58.6%, as calculated with Equation (4).

%m_d_ = (m_i_ − m_d_)/m_i_ (5)

m_i_ is the composts initial mass and m_d_ is the mass of compost dried for 24 h at 100 °C.

Sample films were cut into 2 × 2 cm fragments. 45 fragments of each PLA were buried in 600 mL of compost. At different time during biodegradation experiments, 2 fragments were removed and cleaned with distilled water. They were dried for one hour at 60 °C before characterization by melt rheology.

#### 2.1.5. Photo-Ageing by UV Irradiation

Accelerated photo-ageing was performed in an ATLAS SEPAP 12/24 oven in presence of air at 60 °C. This device is equipped with four “medium pressure” mercury sources (Mazda type MA 400), situated in vertical positions at each corner of the chamber. UV-light irradiation was carried out under polychromatic light with wavelengths below 300 nm, filtered by the glass envelope of the sources. Sampled 2 × 2 cm film fragments were irradiated. Films were fixed on aluminum holders on a rotating carousel positioned in the center of the chamber. Samples surface and sources were separated by 20 cm. In this series of experiments, films were analyzed with up to 100 h of UV exposure.

## 3. Results and Discussion

### 3.1. Biodegradation Pathway

The experiment was validated by total biodegradation of cellulose after 43 days. After few days, transparent samples bleached and became brittle (Table 2). Different PLA had the same behavior and split up.

However, PLLA split up quickly, so the compostability rate could be higher for l-isomers than for d-isomers.

### 3.2. Melt Viscoelasticity to Evaluate UV-Ageing and Composting

#### 3.2.1. UV Ageing

During photo-ageing, PLA molecular evolution is characterized by chain scissions assessed by melt rheology at different exposure times.

**Figure 1:**
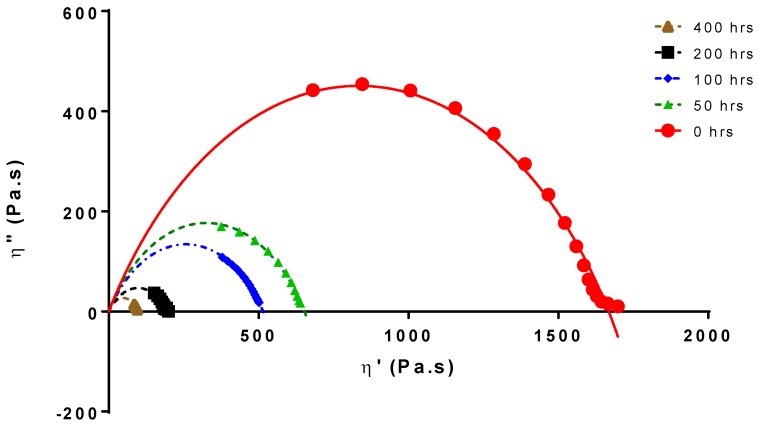
Cole–Cole plot of aged and non-aged PLA4042D.

Chain scission is the cause of the molecular weight and viscosity decreases. Figure 1 shows the Cole-Cole plots for PLA 4042D at different UV exposure times [30].

#### 3.2.2. Molecular Weight Evolution during Composting

**Figure 2:**
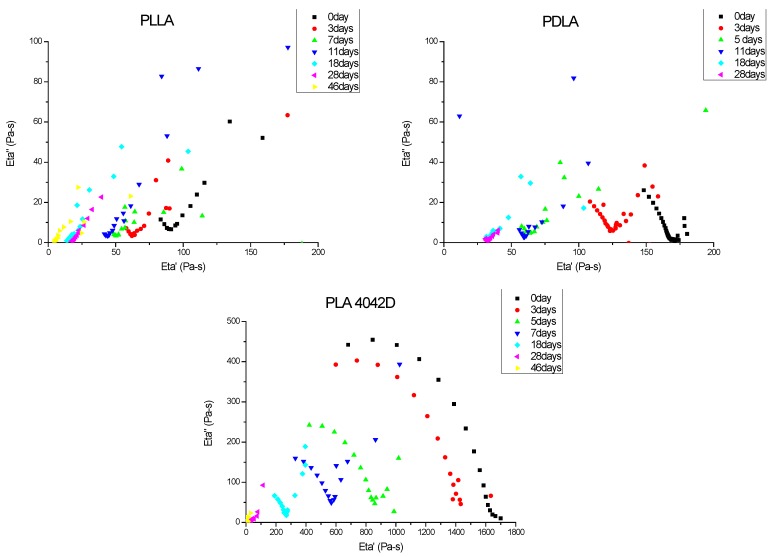
Cole–Cole plots of PLLA, PDLA and PLA4042D during composting (0 to 46 days). Viscoelastic experiments were performed at T = 180 °C.

To observe the effect of biodegradation on the macromolecular structure, the viscoelastic properties of PLA were checked at different burying times. Figure 2 shows the Cole–Cole plots of different PLA samples at different composting times. The zero shear viscosity depends on the molecular weight, so it is higher for initial PLA 4042D than for initial PDLA and initial PLLA (respective values 1630 Pa·s, 165 Pa·s and 91 Pa·s).

Two periods are suspected in the biodegradation mechanism: in the first step, PLA is hydrolyzed and in the second step, micro-organisms digest carbon sources into methane, carbon dioxide and water. In all cases, after few days in compost, the zero shear viscosity decreases until a minimum value ranging between 20 and 30 Pa·s. This critical value of viscosity could correspond to the molecular weight (M_w_) value that the polymer chains must reach to be digested by micro-organisms.

Poly(l-lactic acid) had the lowest molecular weight. Even before composting experiments, the relaxation viscoelastic spectrum shows a superposition of a solid-gel (linear variations of η″ versus η′) with liquid-flow behavior (circular shape). The gel character was more pronounced as the biodegradation time increased. Poly(d-lactic acid) films also exhibited a gel relaxation behavior. This gel character is more important for PLLA, despite a close Newtonian viscosity value. For both polymers, minimum Newtonian viscosity was obtained after 18 days of being buried.

PLA 4042D films showed mainly a viscoelastic liquid-flow behavior (semi-circular) and the gel behavior appeared to increase only after five to seven days of composting. As with the other PLA, a minimum zero shear viscosity was reached, but after a longer time (28 days).

**Figure 3:**
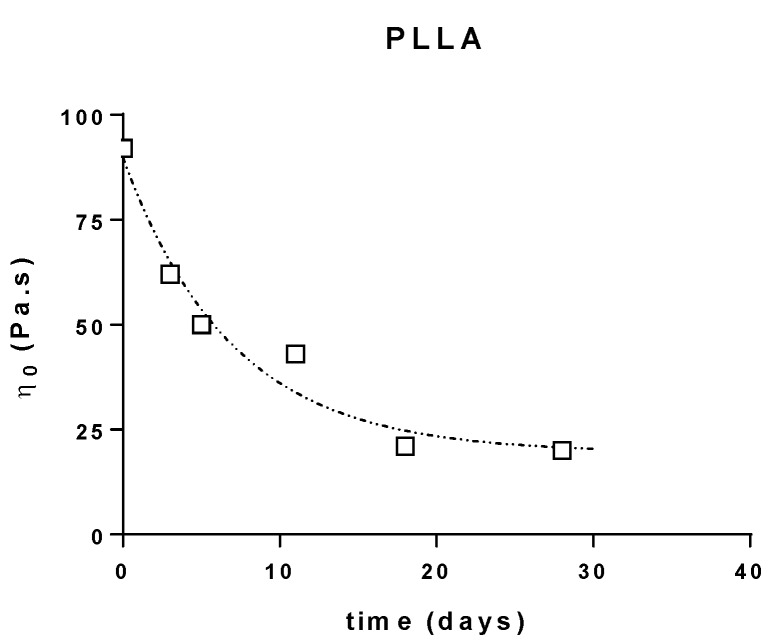

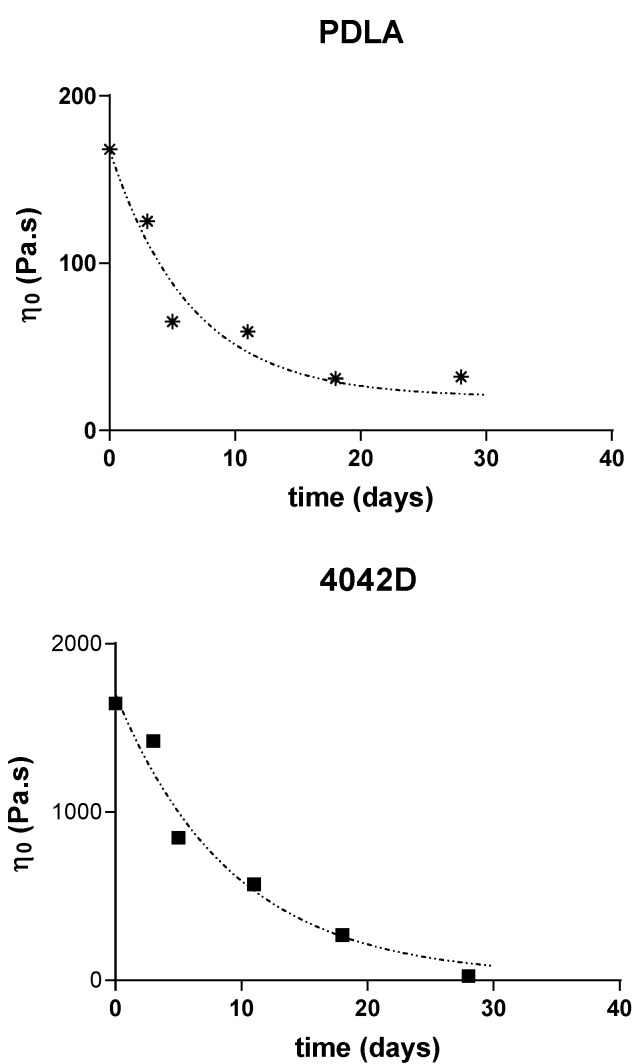
Zero shear viscosity evolution during composting of PLLA, PDLA and PLA4042D.

The variations of the Newtonian viscosities (determined through the extrapolation of the semi-circular component of the Cole–Cole plots) of the different PLA are reported below (Figure 3).

A nonlinear regression using a one phase single exponential decay (dot lines):

Y = (Y_0_ − Plateau) × exp (−kt) + Plateau (6)

This shows that the constant rates for the decrease of molecular weight are quite the same for all the samples (k = 0.12 − 0.15 day^−1^) (R^2^ = 0.9658 (PLLA), 0.9383 (PDLA), 0.9666 (4042D)). The good correlation obtained allowed us to postulate that the microstructure of PLA does not affect its behavior in composting conditions.

The degradation rates showed the same evolution: fast degradation at the beginning, and then a decrease of the viscosity after few days in compost. Moreover, it seems that the same limiting plateau (η_0_ = 20 Pa∙s) is reached for long composting times, which could correspond to a critical molecular weight for the flow regime (Figure 3).

#### 3.2.3. UV Ageing and Its Impact on Biodegradation

**Figure 4:**
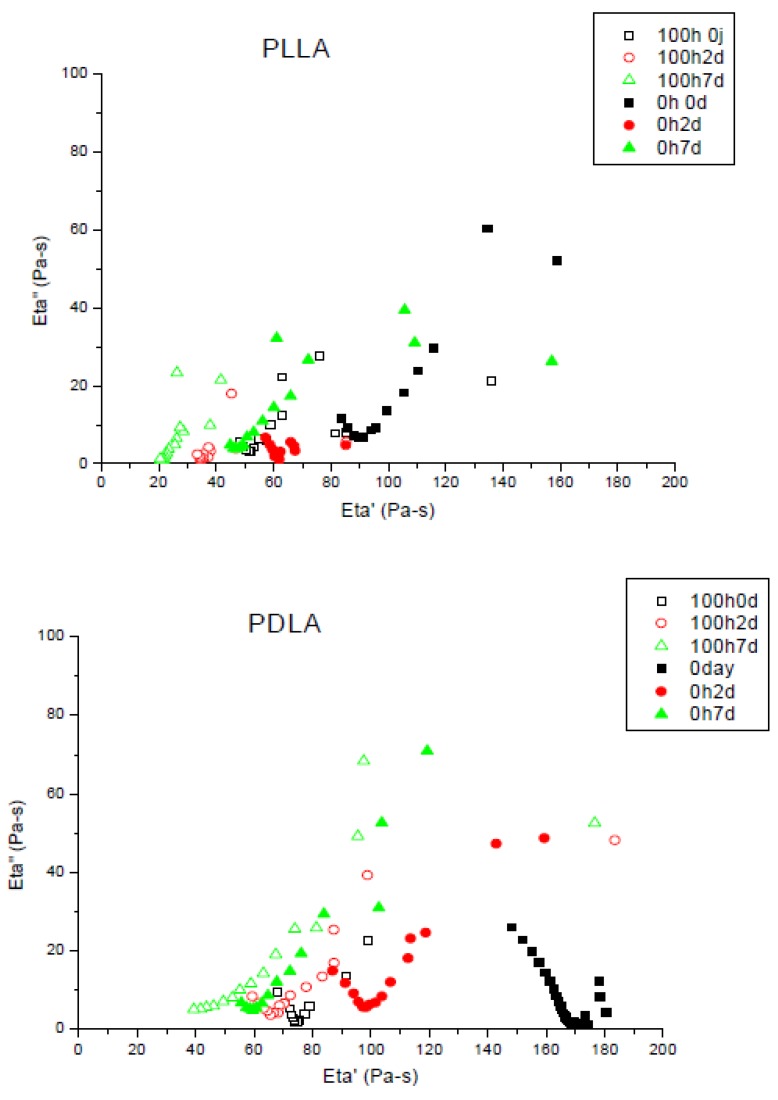

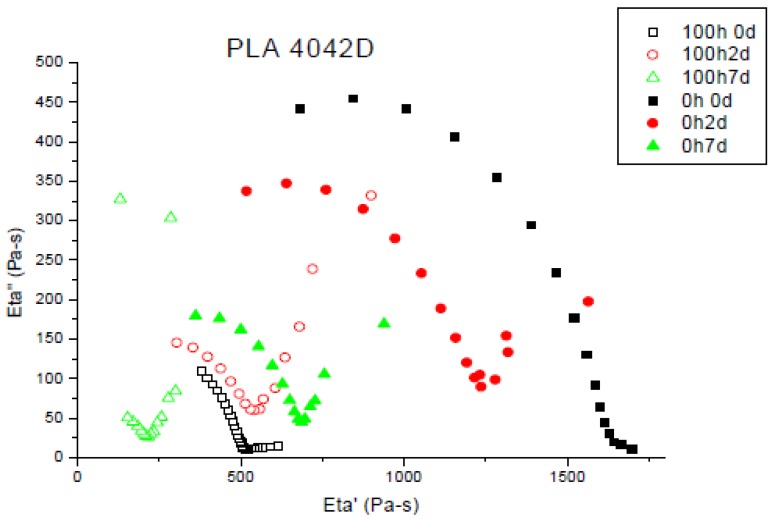
Cole–Cole plots of PLLA, PDLA and PLA4042D non-aged and photo-aged during composting (0 until to 7 days). Unfilled symbols: after 100 h of photo-ageing; filled symbols non-aged. Viscoelastic experiments have been performed at T = 180 °C.

During photo-ageing, M_w_ decreases because of the chain scission mechanism involved. Figure 4 shows the Cole–Cole plots for the initial molecular changes during composting (between 2 and 7 days) of different PLA (L, D and L + D) before UV ageing and after 100 h of UV ageing.

Photo-ageing did not disturb biodegradation, zero shear viscosities also decreased for degraded films. Chain scissions appeared during UV irradiation and have the same effect due to the hydrolysis in composting, it permits the micro-organisms to mineralize the shorter chains of polymer. In the case of PDLA, zero shear viscosities were achieved after seven days buried in compost. Biodegradation was slowed down by the preliminary ageing. Samples with a major L-isomer proportion showed differences between photo-aged and non-aged films. The former showed a higher decrease of viscosity and a higher biodegradation rate. After seven days in compost, non-aged PLA4042D did not achieve a viscosity as low as the polymer photo-degraded before biodegradation experiment.

**Figure 5:**
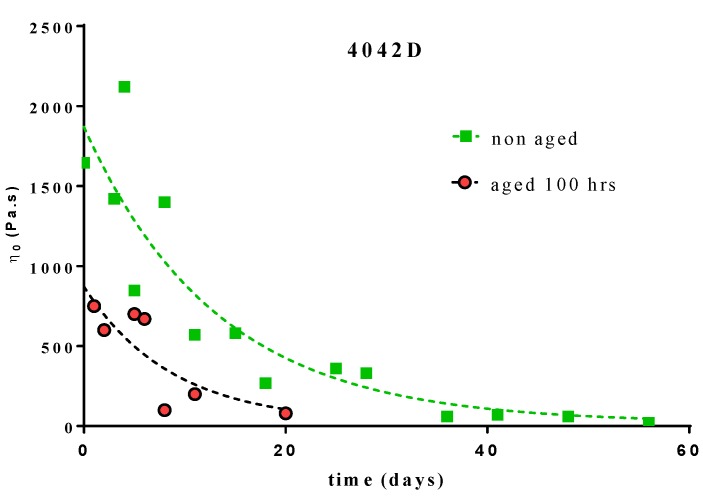
Variation of the Newtonian viscosity versus time of composting for PLA 4042D, 100 h UV aged and non-aged.

The variations of the Newtonian viscosity for the L + D PLA (4042D) during composting are shown on Figure 5.

The single exponential decay (Equation (6)) fit of these variations leads to the following constant rates:

- 100 h UV aged 4042D PLA: k = 0.076 day^−1^ (R^2^ = 0.6849)
- non-aged 4042D PLA: k = 0.115 day^−1^ (R^2^ = 0.8262)

The values of the rate constant were in the same range as previously determined but with a lower correlation (especially for aged PLA). Regarding this correlation, we cannot conclude that UV ageing should significantly influence the kinetic mechanism of PLA bio-assimilation [31]. The same methodology could not be applied to UV aged L and D–PLA because of the low viscosity values of the material after ageing.

#### 3.2.4. FTIR Results

**Scheme 2:**
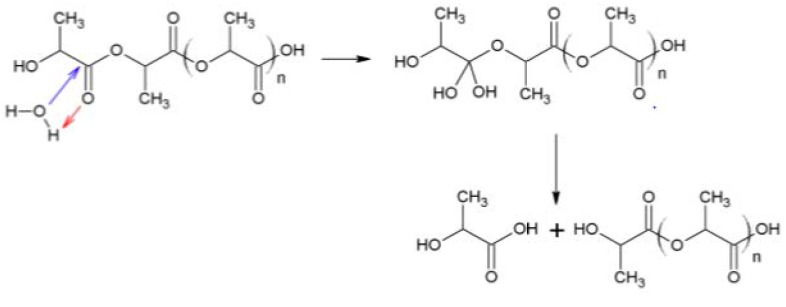
Mechanism of PLA hydrolysis.

**Figure 6:**
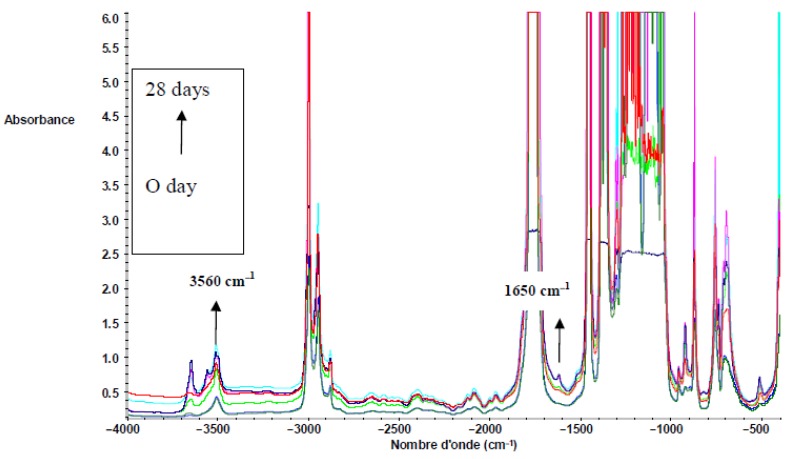
FTIR spectra of PLLA films during composting (0 until to 28 days) in the region of 4000−400 cm^−1^.

Figure 6 shows the FTIR spectra of PLLA in the region from 4000 to 400 cm^−^^1^. Both 3560 (vibration of hydroxyl bonds of lactic acid) and 1625 cm^−1^ (C = C vibration of the vinyl groups) absorption bands appeared during composting, corresponding to lactic acid apparition due to hydrolysis (see Scheme 2). The same evolution was observed for PDLA and PLA 4042D. Hydrolysis of PLA is given below. Vinyl termination is also possible on PLA oligomers.

The most important structural changes were observed in the 1000–600 cm^−1^ region. Three different crystal forms of PLA exist: α, β, and γ crystal forms. The α crystal is the most common form; it is obtained by melt or solution crystallization. The β form is characterized by a 908 cm^−1^ bond and is obtained by drawing at high draw ratios and temperatures. Rarely observed, the γ crystal is another form obtained during epitaxial crystallization.

**Figure 7:**
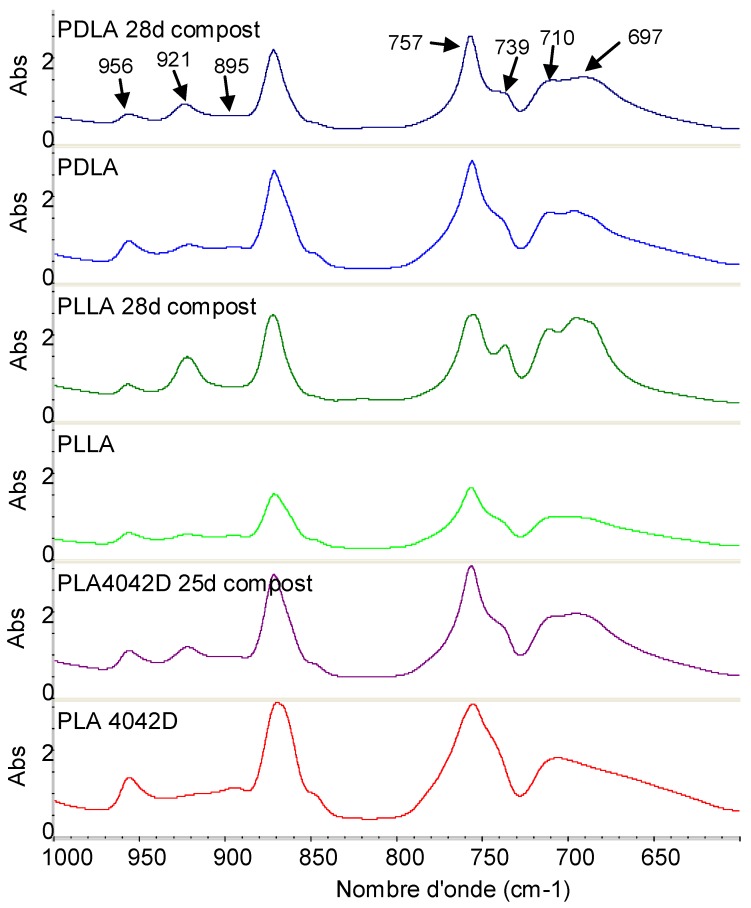
FTIR spectra of PLA films before and after 25 days of biodegradation in the region of 1000−600 cm^−1^.

Figure 7 shows the FTIR spectra of PLLA, PDLA, and PLA4042D in the region of 1000 to 600 cm^−1^. No bands at 908 cm^−1^ were visible suggesting that biodegraded PLLA does not contain a detectable level of β crystals. In Figure 7, the absorption bands at 921, 739 and 697 cm^−1^ are attributed to the crystalline phase and the bands at 956, 895, 757 and 710 cm^−1^ to the amorphous phase [32]. The absorption bands at 921 and 956 cm^−1^ were selected to determine the crystallization changes [33]. The ratio between the absorbance of these specific bands was used to follow the evolution of the crystallization during composting.

**Figure 8:**
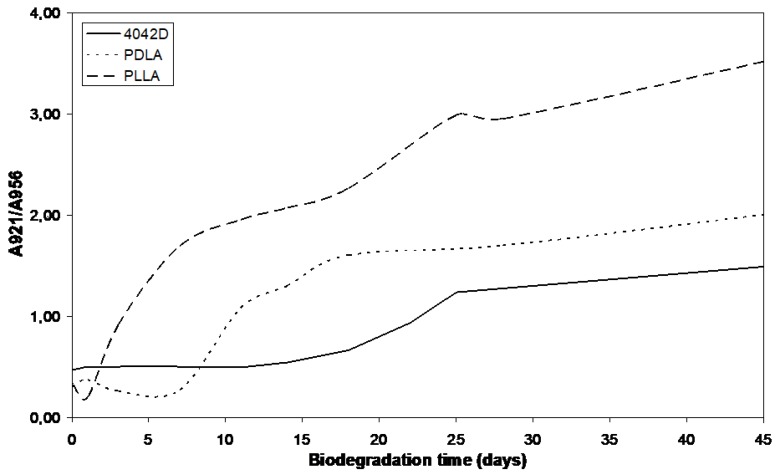
A_921_/A_956_ evolution during composting (0 until to 45 days).

The absorbance intensity of the crystalline phase increased to the detriment of amorphous phase. PLLA and PDLA had close M_w_ values. In Figure 8, PLLA appears more crystallized than PDLA suggesting that L-isomer crystallizes more easily than D-isomer. The crystallization rate was not constant. For PLLA, at the beginning step of composting, the degree of crystallinity increases quickly and after ten days its evolution slows. The first biodegradation step is hydrolysis which induces crystallization of PLA. The rate of this phenomenon decreases because of stiff chains that slow down the penetration of water in crystalline phase, inducing a decrease in the rate of hydrolysis and a deceleration of biodegradation [34]. The same evolution is observed for PDLA but in this case, an induction phase is necessary to observe the crystallinity modification. The induction step was also observed for PLA 4042D. High M_w_ delayed crystals formation as induction step is directly connected with the molecular weight M_w_. Indeed, when M_w_ is high, long chains are not favorable to water permeation and thus to hydrolysis.

The degree of crystallinity still increases after the material has reached the minimum zero-shear viscosity in rheology (18 days for PLLA and PDLA, 28 days for PLA4042D).

## 4. Conclusions

Various stereoisomer of PLA were studied during photo-ageing, and biodegradation in compost. Compression molded films were exposed to UV radiations and buried in compost to evaluate the photo-ageing impact onto the biodegradation properties in the case of PLA.

Chain scissions occurring during UV ageing leads to a decrease of molecular weight observed by melt rheology with zero shear viscosity decrease.

During biodegradation, melt viscoelasticity highlights a chain scission mechanism. The macromolecular chains of PLA must achieve a minimum molecular weight to permit its assimilation by micro-organisms. The crystal phase due to hydrolysis appears and can be followed by FTIR. In all cases of UV aged PLA, zero shear viscosity decreased during composting, as had been observed for non-aged PLA. Finally, regarding the molecular evolution, stereoisomer composition of UV ageing does not radically change the kinetics of the process.

## Figures and Tables

**Table 1:** Polymers physical characteristics.

References	PLA 4042D	PLLA	PDLA
M_w_ (g·mol^−1^)	209,000	138,000	149,000
Polydispersity	1, 8	1, 7	1, 7
T_g_	59 °C	59 °C	59 °C
T_m_	150 °C	180 °C	178 °C
T_c_	-	100 °C	108 °C

**Table 2:** Visual evolution of cellulose and polymer fragments.

Composting Time (Days)	Cellulose	PLLA	PDLA
0	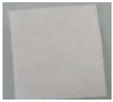	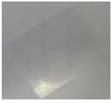	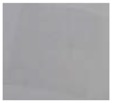
13	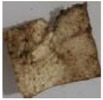	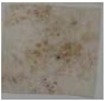	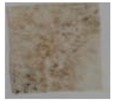
40	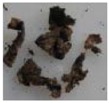	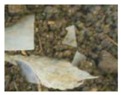	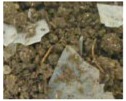
50	Absence of cellulose fragments